# Peptide-Derivatized SB105-A10 Dendrimer Inhibits the Infectivity of R5 and X4 HIV-1 Strains in Primary PBMCs and Cervicovaginal Histocultures

**DOI:** 10.1371/journal.pone.0076482

**Published:** 2013-10-07

**Authors:** Isabella Bon, David Lembo, Marco Rusnati, Alberto Clò, Silvia Morini, Anna Miserocchi, Antonella Bugatti, Sonia Grigolon, Giuseppina Musumeci, Santo Landolfo, Maria Carla Re, Davide Gibellini

**Affiliations:** 1 Department of Experimental, Diagnostic and Specialty Medicine (DIMES), Microbiology Section, University of Bologna, Bologna, Italy; 2 Department of Clinical and Biological Sciences, University of Torino, Orbassano, Torino, Italy; 3 Department of Biomedical Sciences and Biotechnology, University of Brescia, Brescia, Italy; 4 Spider Biotech srl, Colleretto Giacosa, Torino, Italy; 5 Department of Public Health and Microbiology, University of Torino, Torino, Italy; 6 Interuniversity Consortium, National Institute Biostructure and Biosystems (INBB) Roma, Italy; University of Amsterdam, The Netherlands

## Abstract

Peptide dendrimers are a class of molecules that exhibit a large array of biological effects including antiviral activity. In this report, we analyzed the antiviral activity of the peptide-derivatized SB105-A10 dendrimer, which is a tetra-branched dendrimer synthetized on a lysine core, in activated peripheral blood mononuclear cells (PBMCs) that were challenged with reference and wild-type human immunodeficiency virus type 1 (HIV-1) strains. SB105-A10 inhibited infections by HIV-1 X4 and R5 strains, interfering with the early phases of the viral replication cycle. SB105-A10 targets heparan sulfate proteoglycans (HSPGs) and, importantly, the surface plasmon resonance (SPR) assay revealed that SB105-A10 strongly binds gp41 and gp120, most likely preventing HIV-1 attachment/entry through multiple mechanisms. Interestingly, the antiviral activity of SB105-A10 was also detectable in an organ-like structure of human cervicovaginal tissue, in which SB105-A10 inhibited the HIV-1_ada_ R5 strain infection without altering the tissue viability. These results demonstrated the strong antiviral activity of SB105-A10 and suggest a potential microbicide use of this dendrimer to prevent the heterosexual transmission of HIV-1.

## Introduction

HIV induces a life-long infection, which, if untreated, progressively evolves to acquired immunodeficiency syndrome (AIDS) with the ultimate death of the infected patients [**1**]. The World Health Organization has globally estimated that 34 million people were living with HIV-1 at the end of 2011, a year in which 2.5 million of people were newly infected and 1.7 million individuals died with AIDS-related diseases [**2**]. HIV epidemics may produce 20–60 million new infections during the next 15–20 years, particularly in the developing world [**3**]. To tackle the HIV infection, two traditional approaches, represented by the pharmacological treatment and prophylaxis measures, have been proposed [3], [4]. Combined antiretroviral therapy (cART) has dramatically changed the evolution of HIV disease but, unfortunately, has not been able to eradicate the HIV infection [**5]**–[8]. However, the treatment of HIV positive patients with cART suppresses viral replication with a consequent decrease in the viral load that can limit HIV transmission and reduce the number of new infections [**9**]. Preventive socio-behavioural measures, the medical treatment of other sexually transmitted diseases and immunological strategies have also been proposed in an attempt to combat the spread of HIV infections [3], [10], [11]. In particular, several vaccines have been evaluated in trials, but none have been able to induce a sterilizing immune response that prevents HIV transmission and infections in the population [**12**]. The absence of a broadly protective vaccine against the various HIV subtypes indicates that the development of alternative pharmacological approaches to HIV infection prophylaxis may be considered a major avenue for controlling HIV transmission. Because approximately 85% of HIV cases originate from sexual transmission [**13**] and because heterosexual contact represents the major route of infection [**14**], the development of pharmacological topical treatments, that employ so-called microbicides, might be crucial to prevent or reduce the transmission of HIV at the level of the genital mucosa [**15]**–[17]. Mathematical models have predicted that 60%-effective microbicides with 20% coverage could prevent 2.5 millions new infections within three years [**18]**, [19], suggesting that the identification of novel microbicides might play an important role in HIV infection control, pending the development of a functional vaccine.

The viral replication cycle offers several targets for microbicide development [**20**], although both the inhibition of entry and retrotranscription processes are currently considered the most promising targets [**15**]. The strategies to block HIV entry into cells are mainly focused on interference of viral capture, CD4 engagement inhibition, co-receptor binding and gp41 rearrangement resulting in the use of many microbicides, such as polyanions, lectins, monoclonal antibodies, CXCR4, CCR5, CD4 and gp120 binding factors and gp41-fusion inhibitors [**9]**, [21]. In addition, some non-nucleoside (NNRTIs) and nucleoside retrotranscriptase inhibitors (NRTIs) may also be considered microbicides [**22**]. Recently, a tenofovir (TDF) gel that was used during intercourse by women in the Centre for the AIDS Programme of Research in South Africa (CAPRISA) 004 study [**23**] yielded effective results; however, in the VOICE trial, the use of a daily-administered TDF gel failed to reproduce these data [**24**]. Interestingly, similarly controversial results were observed with oral TDF or TDF/FTC once-daily treatments in three separate studies [**25]**–[27], most likely, caused by the different levels of patient adherence to the tested therapy [**28]**, [29]. There are approximately 50 candidate microbicides currently in development, and some of these compounds are in trial phases 2 and 3 [**21**]. Unfortunately, many molecules have not passed the preclinical safety and/or efficacy tests or have failed when clinically tested. An interesting class of molecules with antiviral activity is represented by dendrimers [**30**]. Dendrimers are highly branched macromolecules that possess a poly-functional core associated with multiple functional groups on the surface layer [**30]**–[31]. Dendrimer-based molecules have been described as yielding many potential therapeutic applications and, most importantly, as exhibiting antiviral and antibacterial activities [**32]**, [33]. Notably, dendrimers display polyvalent viral inhibition using multiple repeat domains on a single molecule that can induce a derangement of virus/cell surface and interaction. Sulfonated polylysine dendrimers have shown anti-HSV and antiretroviral activities; indeed, the SPL7013 compound, which is the most active of these dendrimers, has been proposed as a candidate topical microbicide. Interestingly, SPL7013 has exhibited anti-HIV activities *in vitro* [**34]**, [35], *ex vivo* [**36**] and in a macaque challenge study [**37**].

In the present study, we analyzed the anti-HIV activity of the SB105-A10 dendrimer synthetized on a lysine core, which exposed four 9-mer peptide chains [**38**] that have been previously demonstrated to block viral attachment and entry of different viruses [**39]**–[41]. In this investigation, we tested the antiretroviral activity of the SB105-A10 dendrimer on the replication of HIV-1 X4 and R5 strains to determine whether this molecule may be considered a novel compound with anti-HIV properties.

## Materials and Methods

### Cell cultures

The study is in accordance with the provisions of the Declaration of Helsinki and St Orsola-Malpighi Hospital, Bologna, Italy. Peripheral blood samples were collected from healthy blood donors during their routine laboratory analysis at Blood Bank, S.Orsola-Malpighi Hospital, Bologna in according to the rules established by Italian Law (Legislative Decree 03-03-2005, published in G.U. n. 85, 13.04.2005). No approval from Ethical Committee was requested because all blood samples were anonymous and could not related to any blood donor. The PBMCs were separated from peripheral blood samples using a Ficoll gradient (Ficoll-Histopaque, Pharmacia, Uppsala, Sweden) and were seeded in RPMI1640 plus 10% FCS and 2 mM L-glutamine at 5×10^5^ cells/ml. The PBMCs were activated by PHA (5 µg/ml; Sigma, St Louis, MO, USA) plus IL-2 (10 U/ml; Pierce, Rockford, IL, USA) treatment for three days. The medium was replaced every three days with fresh medium (RPMI1640+10%FCS, 2 mM L-glutamine and 10 U/ml IL-2).

### Virus stocks

Sixteen HIV-1 X4 or R5 strains were selected for the experiments. HIV-1_IIIb_ and HIV-1_ada_ are classical laboratory X4 and R5 strains, respectively. Six additional HIV-1 reference strains (ARV-2, RU132, SE9173, MP535, CBL-4 and BaL) were obtained from NIBSC (NIBSC, London, UK), whereas eight HIV-1 isolates were achieved from naïve or cART-treated HIV positive subjects (**Table 1**). Reference HIV strain stocks were prepared in C8166 cells (HIV-1_IIIb_) or in activated PBMCs [**42**] whereas the HIV-1 primary viral isolates from eight HIV-infected subjects were obtained using a co-culture technique, as described previously [**42**]. All of the viral stocks were titrated using an HIV-1 gag p24 antigen ELISA kit (Biomerieux, Marcy L′Etoile, France) at 1000 ng/ml of the HIV-1 gag p24 protein. Viral tropism of HIV-1 primary viral isolates from eight HIV-infected subjects was evaluated by genotypic methods as previously described [**43**].

**Table 1:** HIV-1 strains.

HIV-1 strains	HIV-1 subtype	HIV-1 tropism
HIV-1_IIIb_	B	X4
HIV-1_Ada_	B	R5
HIV-1_ARV-2_	B	R5/X4
HIV-1_RU132_	G	R5
HIV-1_SE9173_	J	R5
HIV-1_MP535_	K	R5
HIV-1_CBL-4_	D	X4
HIV-1_BaL_	B	R5
HIV-1_VNBR5N_ *	B	R5
HIV-1_AMBR5N_ *	B	R5
HIV-1_ACBR5N_ *	B	R5
HIV-1_MCBR5N_ *	B	R5
HIV-1_CLBX4T_ **	B	X4
HIV-1_ARR5×4T_ **	B	R5/X4
HIV-1_FLR5×4T_ **	B	R5/X4
HIV-1_CSX4T_ **	B	X4

* HIV-1 isolated from HIV-1 positive naive patients.

** HIV-1 isolated from cART-treated HIV-1 positive patients.

### Dendrimers

**Figure 1:**
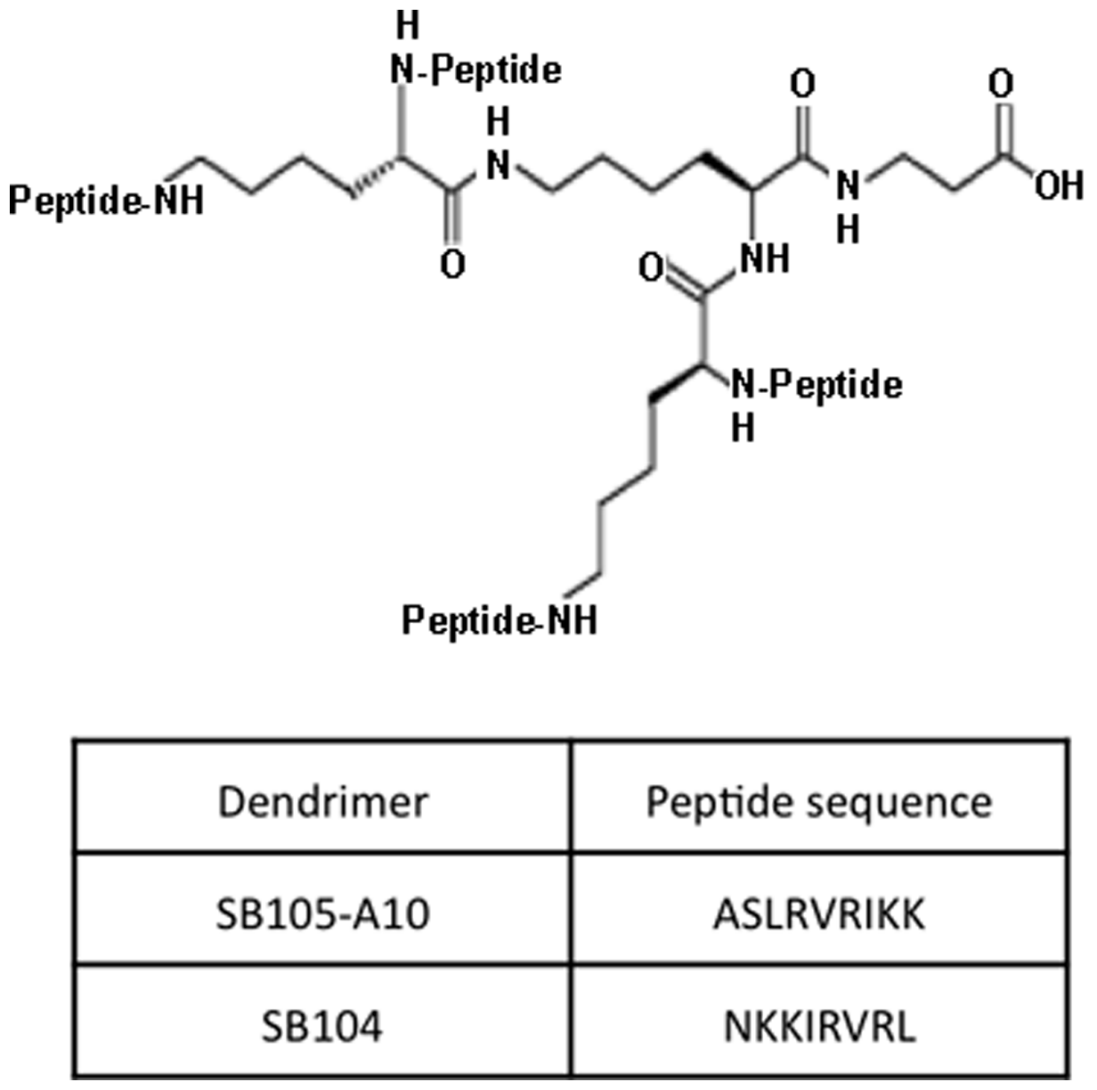
Structure of SB105-A10 and SB104 dendrimers. The general structure of peptide is indicated. These dendrimers were synthesized by the addition of four identical short peptide chains to a tetrameric lysine central core. The aminoacid sequence of four peptide chains in SB105-A10 and SB104 is also shown.

SB105-A10 ([H-ASLRVRIKK]_4_ Lys_2_-Lys-β-Ala-OH; **Figure 1**) and SB104 ([H-NKKIRVRL]_4_-Lys_2_-Lys-β-Ala-OH) dendrimers (Spider Biotech, Turin, Italy) were synthesized as described previously [**39]**, [40]. The lyophilized dendrimers with a purity of >95%, as determined by HPLC-UV (Waters, Milford, MA, USA), were solubilized (1 mg/ml) in phosphate buffered saline (PBS) and stored at −80°C until use. FITC-conjugated SB105-A10 (Polypeptide Laboratories France, Strasbourg, France) was solubilized in PBS at 0.5 mg/ml and stored at −20°C until use. A labeled tetrameric peptide, which bore a biotin moiety linked to the lysine core by a 30-atom pegylated spacer, called SB105-A10-PEG-biotin (Polypeptide Laboratories), had a purity of >95% and was employed in surface plasmon resonance (SPR) analysis.

### HIV infection and dendrimer treatment

The HIV-1 strains (100 pg/ml of HIV-1 gag p24) were pre-incubated for 1 hour at 37°C with scalar concentrations (0, 0.1, 1, 5, 10 and 20 µg/ml) of SB105-A10 or SB104, and then added to activated PBMCs that were adjusted to a final density of 1×10^6^ cells/ml for 2 hours at 37°C. After four washes in PBS, the cells were seeded at 5×10^5^ PBMCs/ml into fresh medium with scalar concentrations of SB105-A10 or SB104. One-half of the medium was replaced with fresh medium plus dendrimers at day 4 post-infection (pi). The HIV-1 gag p24 content was determined at days 4 and 7 pi in culture supernatants, using HIV-1 p24 antigen ELISA kit (Biomerieux). The cell viability was evaluated by the Trypan Blue exclusion technique in the presence of scalar concentrations of the dendrimers.

### Attachment assays

Four different assays were performed.

In the pre-attachment assay, activated PBMCs (1×10^6^ cells/ml) were incubated with SB105-A10 (20 µg/ml; 4.2 µM) for 1 hour at 4°C. After removal of the compound by PBS washes, either HlV-1_lllb_ or HlV-1_ada_ (100 pg/ml of HIV-1 p24) was added to activated PBMCs (1×10^6^ cells/ml) and incubated for 1 hour at 4°C. After two washes with PBS, the activated PBMCs were seeded at 5×10^5^ cells/ml into fresh medium containing the dendrimer (20 µg/ml), and the temperature was changed to 37°C. As control, the activated PBMCs were treated with the same protocol without dendrimer. The HIV-1 gag p24 content was determined at day 4 in the culture supernatants, using an HIV-1 p24 antigen ELISA kit (Biomerieux).

In the post-attachment assay, the activated PBMCs (1×10^6^ cells/ml) were incubated either with HlV-1_lllb_ or HlV-1_ada_ (100 pg/ml of HIV-1 p24) for 1 hour at 37°C. After the PBS washes to remove the unbound virus, the activated PBMCs were seeded at 5×10^5^ cells/ml and incubated at 37°C into fresh medium containing SB105-A10 (20 µg/ml; 4.2 µM). As control, the activated PBMCs were treated with the same protocol without dendrimer. The HIV-1 gag p24 content in the culture supernatants was determined at day 4 using an HIV-1 p24 antigen ELISA kit (Biomerieux).

In the attachment assay, SB105-A10 (20 µg/ml; 4.2 µM) was pre-incubated with either HlV-1_lllb_ or HlV-1_ada_ (100 pg/ml of HIV-1 p24) for 1 hour at 4°C. The mixture was added to the activated PBMCs (1×10^6^ cells/ml) and incubated for 1 hour at 4°C to ensure HIV-1 attachment but not entry. After the PBS washes, activated PBMCs were sedeed at 5×10^5^ cells/ml into a medium containing SB105-A10 (20 µg/ml; 4.2 µM) and shifted to a temperature of 37°C. As control, the activated PBMCs were treated with the same protocol without dendrimer. The HIV-1 gag p24 protein content in the culture supernatants was determined at day 4 using an HIV-1 p24 antigen ELISA kit (Biomerieux).

In the dilution experiments, SB105-A10 (20 µg/ml; 4.2 µM) was pre-incubated with either HlV-1_lllb_ or HlV-1_ada_ (100 pg/ml of HIV-1 p24) for 1 hour at 37°C. The sample volume was diluted 50-fold with RPMI 1640 to reduce the free peptide concentrations to a level below that at which the HIV replication would have been significantly inhibited. The diluted mixture was added to the activated PBMCs (1×10^6^ cells/ml) and incubated for 2 hours at 37°C. After two washes, the cells were seeded at 5×10^5^ cells/ml into fresh medium, and HIV-1 p24 protein content in the culture supernatants was determined at day 4 using an HIV-1 p24 antigen ELISA kit (Biomerieux). As control, activated PBMCs were treated with the same protocol without dendrimer.

### Cell-binding assay for the FITC-SB105-A10 peptide

Activated PBMCs (5×10^6^/ml) were incubated for 1 h at 4°C in PBS containing 2% FBS, with increasing concentrations of the FITC-SB105-A10 dendrimer (0, 2.5, 25, 100 and 250 µg/ml). At the end of the incubation period, the cells were extensively washed with PBS, and the activated PBMCs were analyzed by FACScan flow cytometry (Becton-Dickinson, Palo Alto CA). In parallel experiments, the activated PBMCs were pre-incubated with FITC-SB105-A10 dendrimer (25 µg/ml; 5.3 µM) and then washed with PBS containing 2M NaCl, which is a treatment that removes cationic polypeptides from the cell surface HSPGs [**44**]. Alternatively, the activated PBMCs were either incubated with heparinase III (40 mU/ml; Sigma, St Louis, MO, USA) in 20 mM TrisHCl pH 7.5, 0.1 mg/ml BSA and 4 mM CaCl_2_ for 2 h at 37°C or left untreated prior to being assayed for binding between the activated PBMCs and 25 µg/ml (5.3 µM) of FITC-SB105-A10 dendrimer. The samples were analyzed by FACScan flow cytometry.

### Surface plasmon resonance (SPR) assay

SPR measurements were performed on a BIAcore X100 instrument (GE-Healthcare, Milwaukee, WI), using a research-grade SA streptavidin-precoated sensorchip. To study the SB105-A10 interaction with the HIV-1 glycosilated recombinant gp41 ectodomain (amino acids 546-682; NIBSC, UK) and the full-lenght recombinant gp120 glycoprotein (NIBSC), biotinylated SB105-A10 [10 µg/ml in 10 mM HEPES buffer pH 7.4 containing 150 mM NaCl, 3 mM EDTA, 0.005% and surfactant P20 (HBS-EP)] was injected onto the SA sensorchip, allowing the immobilization of 560 resonance units (RU) equal to 0,12 pmol/mm^2^ of the tetrameric peptide. A sensorchip coated with streptavidin alone was used for blank subtraction and to evaluate the non-specific binding. Increasing concentrations of gp41 and gp120 in HBS-EP buffer were allowed to associate with the SB105-A10- or streptavidin-coated surfaces for 3 min and then washed for 10 min to allow their dissociation. As a further control of specificity, bovine serum albumin (BSA) and the glycosylated lectin from *Sambucus nigra* (SNA) (Vector Lab, Burlingame, CA, USA) were injected on the SB105-A10 surface under the same experimental conditions described above. After every run, the sensorchip was regenerated by the injection of 2 M NaCl. The steady-state affinity was calculated by the Biacore X100 evaluation software from the RU values of HIV proteins that were bound at equilibrium to the SB105-A10 surface.

### Cervicovaginal tissue model

The EpiVaginal Tissue Model (VLC-100FT; MatTek Corp., Ashland, MA, USA) was kept in a 24-well plate containing a proprietary growth medium, according to the manufacturer's indications. This in vitro-reconstituted, full thickness vaginal tissue model was formed by a complete, stratified vaginal–ectocervical epithelial layer mixed with Langerhans cells and with an additional fibroblast-containing lamina propria. Each EpiVaginalTM tissue-containing well was treated with 100 µl of topically applied PBS (negative control), PBS containing HIV-1_ada_ (25 ng p24/tissue) or PBS containing HIV-1_ada_ plus the SB105-A10 dendrimer (2 or 10 µg/tissue) for 24 hours. The apical surface of each tissue was washed twice with PBS, and either 50 µl of PBS or PBS plus the SB105-A10 dendrimer (1 or 5 µg/tissue) was added at day 1. The underlying media (2 ml/tissue) was changed every other day, and the tissues were harvested at day 4 pi. The vaginal tissues and culture supernatants were collected for isolation of the HIV-1 DNA. The total DNA was extracted and purified from the vaginal tissues using the DNA easy kit (Qiagen, Hilden, Germany). The HIV-1 DNA content was determined by SYBR Green-based quantitative real-time PCR and normalized as already described [**45**]. The oligonucleotide pairs were *gag* and *pol* gene specific primers previously indicated [**45]**, [46].

### Evaluation of the irritation potential of SB105-A10 on EpiVaginal system

The cytotoxicity of SB105-A10 on the mucous membranes was assessed using the EpiVaginal system and an MTT ET-50 tissue viability assay, followed by analysis of the lactate dehydrogenase (LDH) levels, according to the manufacturer's instructions. SB105-A10 (100 µg/ml; 21 µM) was added to the cell culture insert on top of the EpiVaginal tissue samples and incubated for 1, 4, or 18 hours in duplicate. At the end of the incubation at 37°C, any liquid on top of the EpiVaginal tissue was decanted, and the inserts were gently rinsed with PBS to remove the residual material. Subsequently, the tissues were processed according to the MTT kit protocol (MatTek Corporation) and read using an ELISA plate reader at a wavelength of 570 nm. Tissues, incubated with ultrapure water, were employed as negative controls. Triton X-100 (1%) was used as the positive control. The ET_50_ is the time required to reduce the tissue viability to 50% and was determined using the Prism software (GraphPad Software, San Diego, CA). To analyze the release of LDH from the treated EpiVaginal tissues into the culture medium, an LDH cytotoxicity detection kit (TaKaRa Bio Inc, Japan) was utilized according to the manufacturer's protocol.

To evaluate the inflammatory response, the EpiVaginal tissues were treated with the dendrimeric peptide SB105-A10 (100 µg/ml; 21 µM) for different exposure times of 1, 4, and 18 h, as reported previously. After incubation, the concentration of interleukin-1 alpha (IL-1α) in the culture medium was measured using an IL-1α ELISA kit (Bender Medsystem, Wien, Austria). The concentration of IL-1α was calculated by interpolation of a standard calibration curve. IL-1α was chosen as a marker of pro-inflammatory activity as suggested by the technical data sheet from MatTek Corporation.

### Statistical analysis

The results were expressed as the means ± standard deviations (SD) of at least three separate experiments performed in duplicate. A two-tailed Student's test was used for the statistical comparison.

## Results

### SB105-A10 inhibits HlV-1_IIIb_ and HlV-1_ada_ replication in activated PBMCs

**Figure 2:**
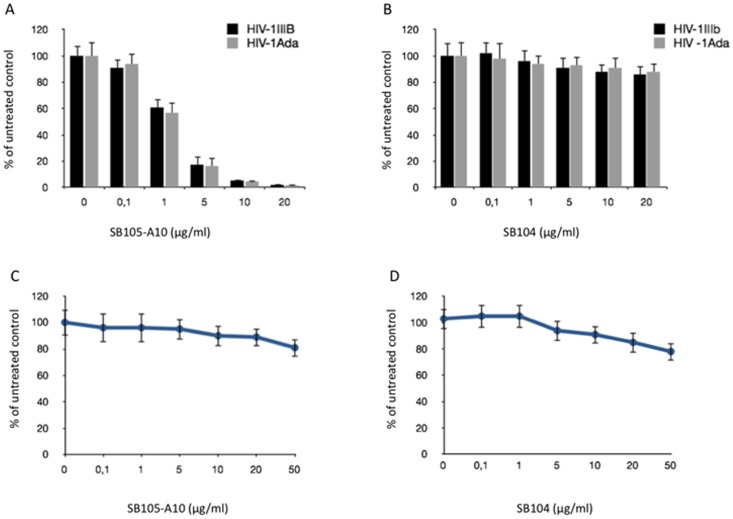
SB105-A10 inhibits HIV-1_IIIb_ and HIV-1_ada_ replication in activated PBMCs. HIV-1_IIIb_ and HIV-1_ada_ were pre-incubated for 1 hours at 37°C with different concentrations (0, 0.1, 1, 5, 10 and 20 µg/ml) of SB105-A10 (**A**) or SB104 (**B**) and challenged with activated PBMCs for 2 hours at 37°C. HIV replication was monitored by HIV-1 p24 ELISA and values were determined from cell culture supernatants at day 7 pi. Data were expressed as the means ± standard deviations (±SD) of HIV-1 gag p24 amount relative to untreated controls (set to 100%). Three experiments in duplicate were performed. The last two panels (**C** and **D**) show that the two dendrimers are not toxic for PBMCs at concentrations tested as documented by trypan blue technique. Data were expressed as the means (±SD) of viable cells relative to untreated controls (set to 100%) obtained from three independent experiments in duplicate. * p<0.05.

In the first set of experiments, the antiviral effects of SB105-A10 on the HIV-1 replication were evaluated. SB105-A10 was challenged with the HIV-1 X4 and R5 laboratory strains, represented by HIV-1_IIIb_ and HlV-1_ada_, respectively. Scalar concentrations of SB105-A10 (0, 0.1, 1, 5, 10 and 20 µg/ml) were incubated with either HIV-1_IIIb_ or HlV-1_ada_ (100 pg of p24/ml) and, after 2 hours at 37°C, this mixture was added to the activated PBMCs. The HIV-1 p24 protein content was evaluated in the culture supernatants at days 4 and 7 post-infection (pi). A significant decrease in the p24 protein was detected in a SB105-A10 concentration-dependent way. The determination of the IC_50_ yielded values of 1.42 µg/ml (0.3 µM) and 1.28 µg/ml (0.27 µM) at day 7 pi in the samples that were infected with HIV-1_IIIb_ and HlV-1_ada_, respectively (**Figure 2A**).

When the same experimental protocol was performed by substituting SB105-A10 with SB104, which is a dendrimer possessing a same net positive charge (four basic amino acids) but a different sequence (NKKIRVRL) in the external peptide chains, no significant decrease in the levels of the p24 protein amount was observed (**Figure 2B**). To rule out a relationship between the HIV-1 p24 decrease and the SB105-A10-related cytotoxicity, scalar concentrations of SB105-A10 (0.1, 1, 5, 10, 20 and 50 µg/ml) were assayed on PBMCs, and the cell viability was analyzed by the trypan blue exclusion technique. The viability of both SB105-A10- and SB104-treated cells was not significantly affected when compared with the untreated cell cultures (**Figure 2C-D**) and yielded a CC_50_ value >50 µg/ml (>10.5 µM).

### SB105-A10 exhibits antiretroviral activities in the HIV-1 reference and patients' isolated strains

**Figure 3:**
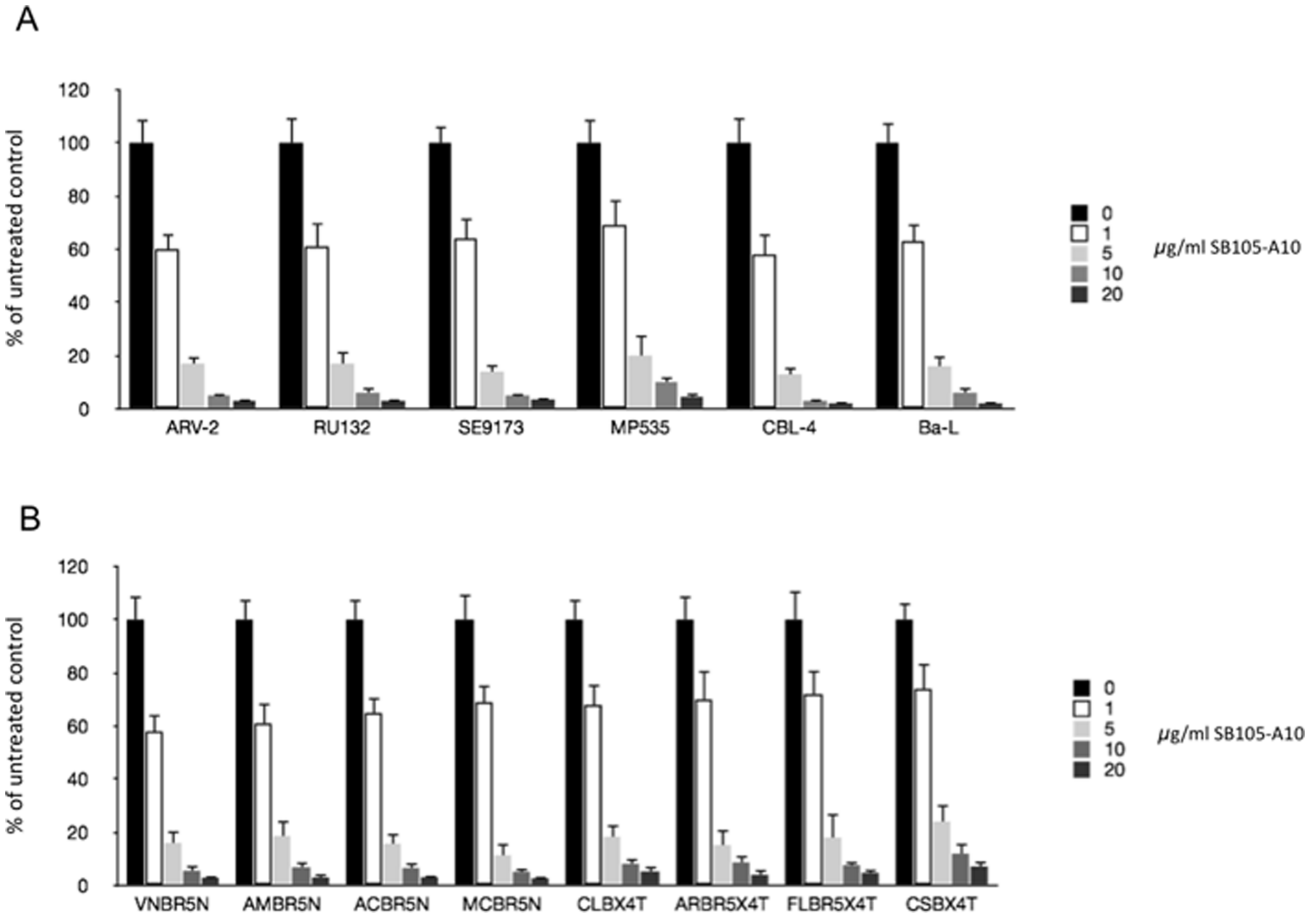
SB105-A10 treatment inhibits the HIV replication of HIV-1 reference strains and isolates from HIV-1 positive pateints. Different concentrations (0, 0.1, 1, 5, 10 and 20 µg/ml) of SB105-A10 were added to HIV-1 reference (**A**) and isolated (**B**) strains (100 pg/ml p24) for 1 hours at 37°C and then added to activated PBMCs. After two hours, several PBS washings were carried out and p24 determination was performed at day 7. Significant decreases of HIV-1 p24 amount were detected in infected samples treated with 5, 10 and 20 µg/ml of SB105-A10 with respect to the untreated controls. Data were expressed as the means (±SD) of HIV-1 gag p24 amount relative to untreated controls (set to 100%) obtained from three independent experiments in duplicate. * p<0.05.

Reference HIV-1 R5 (RU132, SE9173, MP535, BaL), dual tropic X4/R5 (ARV-2) and X4 tropic (CBL-4) strains (**Table 1**) were challenged with scalar concentrations of SB105-A10 (0.1, 1, 5, 10 and 20 µg/ml) in the activated PBMCs. The higher concentrations of SB105-A10 (5, 10, and 20 µg/ml) significantly (p<0.05) decreased the HIV-1 p24 content in the cellular supernatants at day 7 pi, irrespective of the HIV-1 strain employed (**Figure 3A**). Subsequently, eight HIV strains were isolated and purified from the HIV-positive patients. Four of the subjects were naïve patients, whereas the remaining four were patients exhibiting therapeutic failure and resistance against certain protease and reverse transcriptase inhibitors. These viral strains were sequenced and characterized for their cellular tropism. All naïve patients were infected with R5 viral variants, two of the cART-treated patients were infected with the X4/R5 dual tropic strains, and the remaining two of the cART-treated patients harboured X4 strains (**Table 1**). The activated PBMCs were challenged with these HIV-1 isolated from HIV-1 positive patients. Similarly to the experiments using HIV-1 reference strains, the higher concentrations of SB105-A10 (5, 10 and 20 µg/ml; p<0.05) inhibited the viral replication, as demonstrated by the HIV-1 gag p24 ELISA assay (**Figure 3B**) at day 7; in contrast, SB104 did not exhibit any significant anti-retroviral effects (data not shown). The determination of the IC_50_ yielded values between 1.26 (0.27 µM) and 2.1 µg/ml (0.44 µM) among all the tested reference and patients' isolated HIV-1 strains.

### Analysis of the inhibitory mechanism of SB105-A10

**Figure 4:**
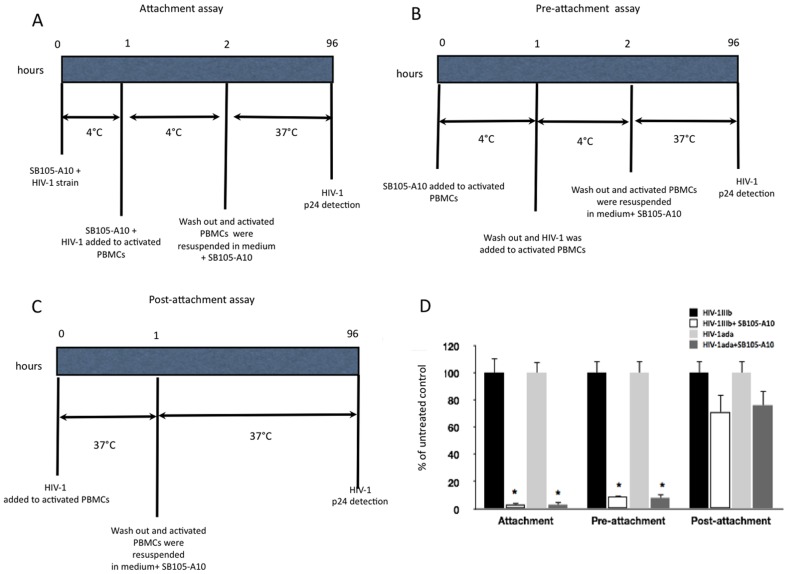
Investigation of SB105-A10-related antiviral mechanisms in activated PBMCs infected by HIV-1_IIIb_ or HIV-1_ada_ strains. Attachment, pre-attachment and post-attachment assays were carried out as described in panel **A**, **B** and **C** respectively. Significant decreases of HIV-1 gag p24 in the cellular supernatants were detected at day 4 pi, in SB105-A10-treated samples with respect to untreated controls in pre-attachment and attachment assays (panel **D**). Data were expressed as the means (±SD) of HIV-1 gag p24 amount relative to untreated controls (set to 100%) obtained from three independent experiments in duplicate. *p<0.05.

To determine the stage of the viral replication cycle at which SB105-A10 interferes with the infection, time-binding assays (**Figure 4** **A-C**) were performed using activated PBMCs that were challenged with either HIV-1_IIIb_ or HIV-1_ada_.

In the “attachment assay” (**Figure 4A**), SB105-A10 was pre-incubated with either HIV-1_IIIb_ or HIV-1_ada_ strains for 1 hour at 4°C, and this mixture was added to the activated PBMCs and incubated for 1 hour at 4°C. This protocol facilitated the eventual attachment of the virus to the cell membrane but did not allow for viral entry. After washes with PBS, the activated PBMCs were shifted to a temperature of 37°C, and the infection was allowed to proceed. The analysis of the HIV-1 p24 protein content, in the cell culture supernatants at day 4 pi, demonstrated a strong neutralizing effect on infections of HIV-1 strains (**Figure 4D**) thus indicating that SB105-A10 might act on the viral attachment to the cell membranes of the activated PBMCs.

In the “pre-attachment assay,” (**Figure 4B**) the activated PBMCs were treated with SB105-A10 for 1 hours at 4°C to allow for interaction of the dendrimer with the cell surface. After several washes, the activated PBMCs were incubated with HIV-1_IIIb_ or HIV-1_ada_ for 1 hour at 4°C, washed, and shifted to a temperature of 37°C. The analysis revealed that, at day 4 pi, and under these experimental conditions, SB105-A10 consistently inhibited the infections by both HIV-1 strains (**Figure 4D**), indicating that the activity of the dendrimer might also depend on its interaction with the PBMC membrane surface.

The “post-attachment assay” (**Figure 4C**) was performed to evaluate whether SB105-A10 could also prevent infection by the cell-bound virus. The activated PBMCs were incubated with either HIV-1_IIIb_ or HIV-1_ada_ for 1 hour at 37°C and were repeatedly washed to remove the unbound virus. Subsequently, SB105-A10 was added to activated PBMCs, and HIV-1 p24 was analyzed at day 4 pi. The inhibition of the HIV-1 infection was not detected by this experimental approach, suggesting that the antiviral effect of SB105-A10 might be related to the interference of HIV adsorption and/or entry (**Figure 4D**).

### SB105-A10 interacts with HIV envelope proteins

**Figure 5:**
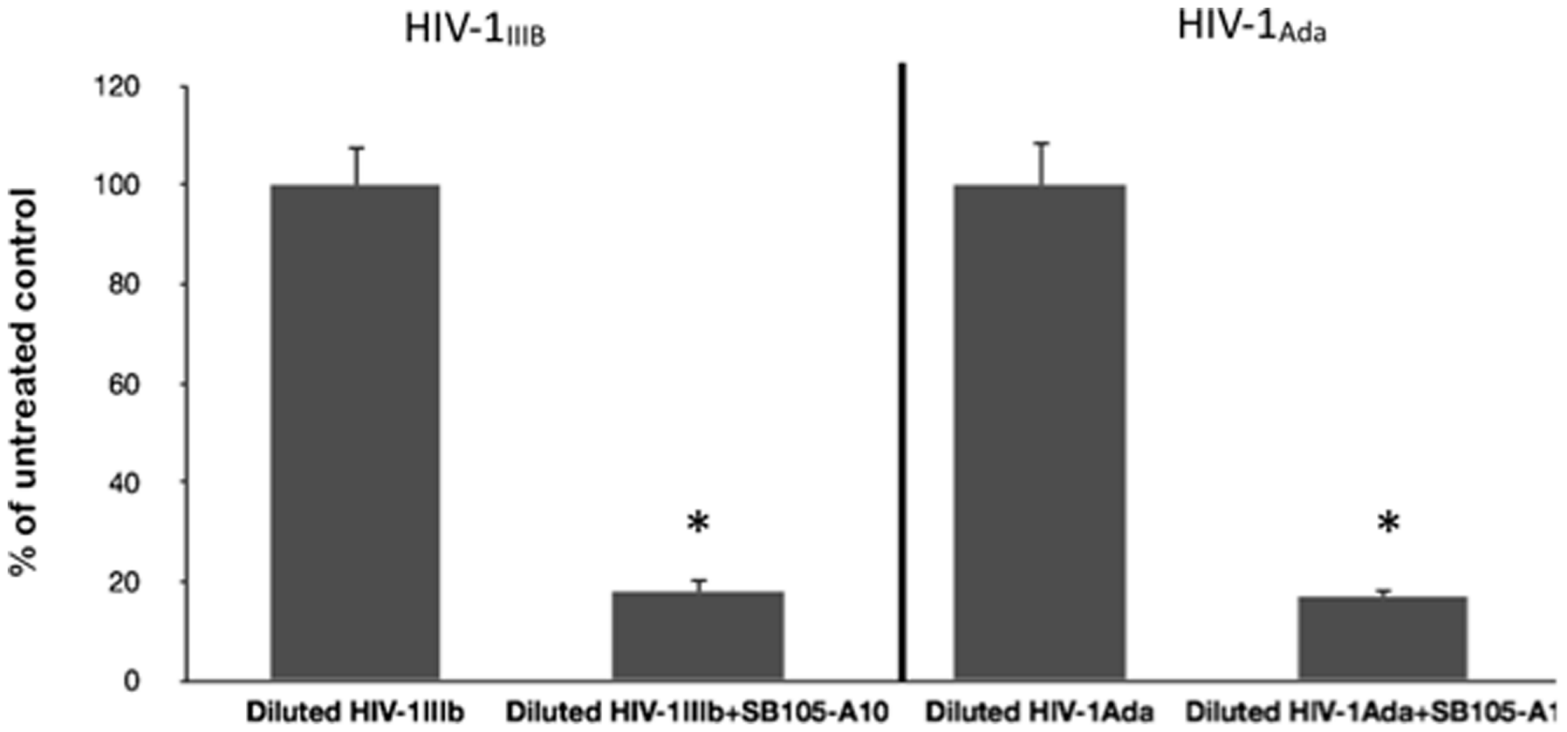
Pre-incubation of SB105-A10 either with HIV-1_IIIb_ or HIV-1_ada_ strains inhibits the viral infectivity in dilution assay. HIV-1 strains were incubated for 1 hour at 37°C with SB105-A10 (20 µg/ml; 4.2 µM). Following this incubation the samples were diluted 50-fold to reduce dendrimer concentrations to a level below that at which SB105-A10 significantly inhibits HIV replication and challenged with activated PBMCs. Significant decrease of HIV-1 p24 amount was detectable at day 4 pi. Data were expressed as the means (±SD) of HIV-1 p24 amount relative to untreated controls (set to 100%) for each HIV-1 strain obtained from three independent experiments in duplicate. * p<0.05.

We also investigated the possibility that at least some of the antiviral effects might be related to a direct interaction between the HIV particles and SB105-A10. Either HIV-1_IIIb_ or HIV-1_ada_ was incubated with 20 µg/ml (4.2 µM) of SB105-A10 for 1 hour at 4°C or 37°C. Following incubation, these mixtures were diluted 50-fold in a medium to reduce the concentration of dendrimer to below the concentration capable of preventing the HIV infection; the diluted mixtures were subsequently added to the activated PBMCs. The analysis of HIV-1 p24 revealed that SB105-A10 significantly reduced the level of HIV-1 infection, suggesting the direct activity of SB105-A10 on the virion structures (**Figure 5**).

**Figure 6:**
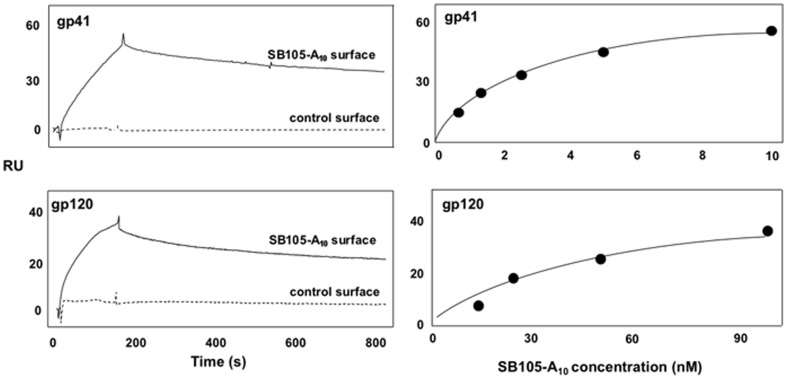
SPR analysis of the interaction of SB105-A10 with HIV-1 gp41 and gp120 proteins. Left panels: blank-subtracted sensorgrams showing the binding of gp41 ectodomain (aminoacids 546–682; 10 nM) and full-lenght recombinant gp120 glycoprotein (100 nM) to the sensorchips containing streptavidin and biotinylated SB105-A10 (straight line) or streptavidin alone (dotted line). Right panels: saturation curves of the binding of gp41 and gp120 to immobilized SB105-A10 peptide. The saturation curves were obtained using the values of RU bound at equilibrium after the injection of different concentrations (0.61, 1.25, 2.5, 5.0, 10 nM for gp41 and 12.5, 25, 50, 100 nM, for gp120). The χ^2^ values of the fitting of the saturation curves were equal to 2.6 and 4.3 for gp41 and gp120 respectively.

To substantiate the hypothesis that SB105-A10 inhibits the HIV infection by sequestering the virus in the extracellular environment, we investigated the dendrimer capacity to bind HIV-1 gp41 and/or gp120. To this end, SB105-A10 was immobilized onto a SPR sensorchip, and evaluated for its capacity to bind the two viral proteins when injected in the fluidic system of a BIAcore X-100 apparatus. As displayed in **Figure 6** (left panels), the ectodomain of gp41 (amino acids 546–682) and full-lenght gp120 interacted with SB105-A10. The specificity of the bindings was proven by the observations that: *i)* both the HIV proteins did not bind to a control surface containing only streptavidin; *ii)* one eukaryotic protein (BSA) and one glycosylated plant protein (SNA) poorly bound the SB105-A10 surface (less than 5 RU and 34 RU, respectively) when injected at 300 nM in the same experimental conditions used for the HIV proteins (data not shown). Dose response experiments allowed us to generate saturation curves (**Figure 6**, right panels), from which dissociation constant (K_d_) values for the two interactions were calculated. In detail, SB105-A10 binds to gp41 with an affinity that is 10 times higher than that for gp120 (K_d_ equal to 3.9 nM and 46 nM, respectively). These results suggest that the dendrimer might interact with the viral envelope, most likely altering the virus attachment and entry.

### SB105-A10 binds to heparan sulfates (HS) on activated PBMC membranes

**Figure 7:**
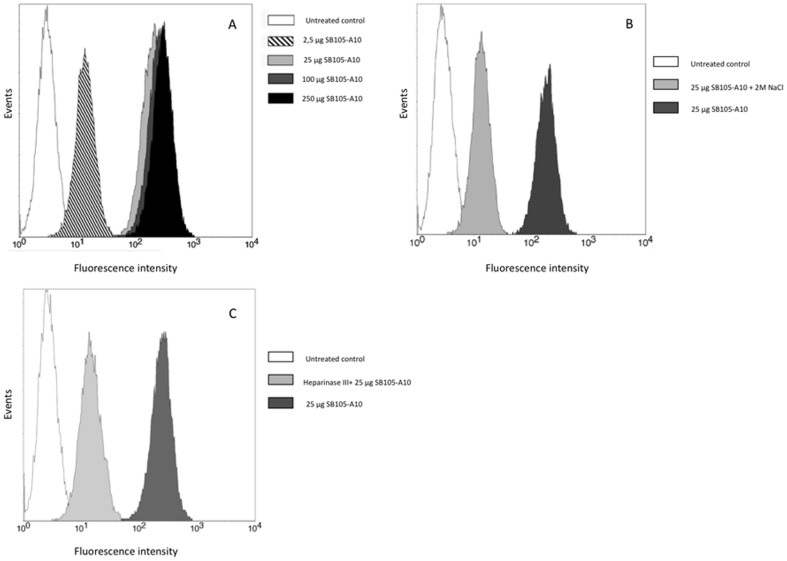
Flow cytometry analysis of binding between SB105-A10 and heparan sulphates. Typical experiments were shown. (**A**), Different concentrations of FITC-conjugated SB105-A10 (from 0 to 250 µg/ml) were assayed for 1 hour at 4°C on activated PBMCs (5×10^6^/ml). The saturation was achieved at 25 µg/ml. (**B**), Activated PBMCs (5×10^6^/ml) were treated with 25 µg/ml of FITC-conjugated SB105-A10 for 1 hour at 4°C and then washed with PBS containing 2 M NaCl, a treatment known to remove cationic polypeptides from cell surface HSPGs. (**C**), Activated PBMCs (5×10^6^/ml) were incubated with heparinase III for 2 h at 37°C or left untreated before the binding assay with 25 µg/ml of FITC-conjugated SB105-A10. Both heparinase III and 2 M NaCl treatments reduce but not abolish the specific fluorescence signal suggesting an interaction between SB105-A10 and cell membrane that is not related to HSPGs.

In addition to the direct activity of SB105-A10 on the envelope glycoproteins, the pre-attachment assay indicated that the dendrimer might inhibit HIV infection through direct binding with molecular targets on the cell membrane. Moreover, the effects of SB105-A10 on the replication of viruses such as HSV, HPV, RSV and HCMV have demonstrated that this molecule binds to HSPGs [**39]**–[41]. Because HSPGs play an important role in HIV adsorption and entry, we evaluated whether the binding between SB105-A10 and HSPGs was also detectable in activated PBMCs. A flow cytometry analysis performed using increasing concentrations of the dendrimer (**Figure 7**), yielded a net increased fluorescence following the FITC-SB105-A10 exposure with a binding saturation at 25 µg/ml (5.3 µM). This interaction was partially inhibited by pre-treatment with heparinase III, which cleaved the HSPGs and significantly decreased, but did not eliminate, the positive signal. Moreover, the activated PBMCs treated with FITC-SB105-A10 and further washed with 2M NaCl, which is a treatment that removes cationic peptides from HSPGs in the cell membrane [**44**], displayed decreased fluorescence, as measured by flow cytometry; however, the binding of FITC-SB105-A10 with HSPGs was not abolished, thus suggesting that the dendrimer might also recognize additional receptors on the cell surface.

### SB105-A10 inhibits the HIV-1 infection of cervicovaginal tissues

**Figure 8:**
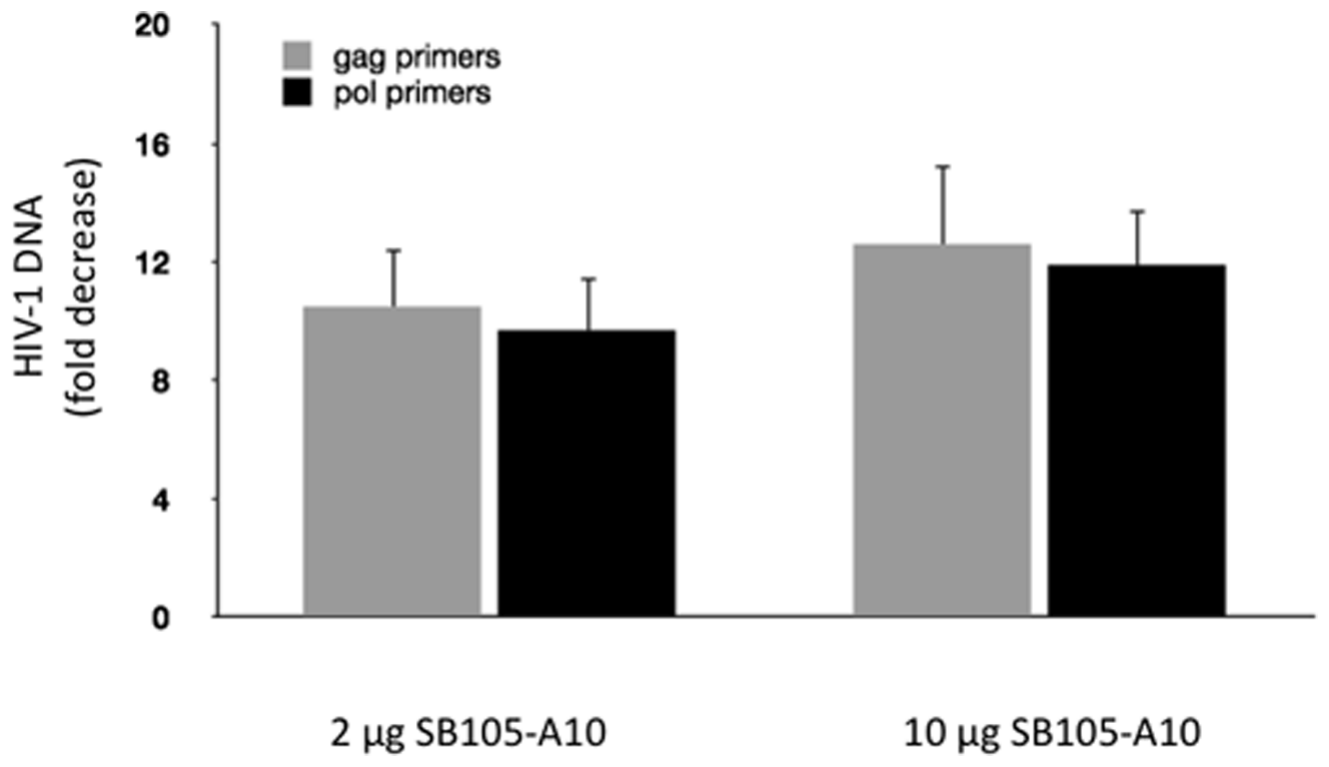
Determination of HIV-1 proviral DNA content in Epivaginal tissue infected by HIV-1_ada_. EpiVaginal™ tissues were challenged with HIV-1_ada_ (25 ng/ml) plus the SB105-A10 dendrimer (2 or 10 µg/tissue) for 24 hours, and total DNA was extracted and purified from vaginal tissues at day 4 pi. HIV-1 DNA burden was determined by SYBR Green-based quantitative real time PCR using either HIV-1 *gag* or *pol* gene-specific primer pairs [**45]**, [46]. Data are expressed as the fold decrease (±SD) of the DNA proviral content in dendrimer-treated cell culture with respect to DNA proviral amount determined in untreated cell cultures, obtained from two independent experiments in quadruplicate.

To investigate the possible use of SB105-A10 as a microbicide, we also examined its role in preventing the HIV-1 infection, using a biologically organotypic model of cervicovaginal epithelial tissue. To determine whether SB105-A10 acts as an antiretroviral agent in vaginal tissues, cervico-vaginal samples were infected apically by HIV-1_ada_ (100 ng HIV-1 p24/tissue) either with or without the dendrimer (2 or 10 µg/tissue) and were incubated for 24 hours; subsequently, the HIV-1 proviral DNA levels were determined by quantitative real-time PCR at day 4 pi using two oligonucleotide pairs specific for HIV-1 gag and pol genes. The samples that were treated with SB105-A10 exhibited approximately 10-fold decrease in their viral DNA burden compared with the untreated HIV-1-infected vaginal tissue, suggesting that SB105-A10 inhibits the HIV-1 infection of a biologically relevant human cervicovaginal tissue model (**Figure 8**). Because the EpiVaginal tissue is ideally suited to predict the vaginal toxicity of novel microbicides we also evaluated the irritation and inflammatory potential of SB105-A10. Briefly, 100 µg/ml (21 µM) of SB105-A10 was applied to the apical surface at the air-tissue interface for 1, 4, and 18 hours at 37°C, and the tissues were subsequently analyzed for (i) the reduction of tetrazolium salt (MTT), to study the metabolic activity of the living cells; (ii) LDH release, to measure the accumulation of dead cells; and (iii) the release of IL-1 α, to evaluate the inflammatory activation of cells (see the Materials and Methods for further details). As reported in **Table 2**, SB105-A10 was not cytotoxic, and the time required to reduce tissue viability to 50% (ET_50_) was greater than 18 hours. No significant difference in the release of the cytoplasmic enzyme LDH was observed between SB105-A10-treated and untreated tissues. Finally, our results indicate that SB105-A10-exposed human-derived vaginal epithelial cells do not exhibit significant differences in the levels of the pro-inflammatory cytokine IL-1α, (**Table 2**), compared with the untreated samples. Similar results were obtained when a 10-fold higher dose of SB105-A10 was applied to the EpiVaginal tissue (data not shown). Overall, these results indicate that SB105-A10 is neither toxic nor pro-inflammatory to the vaginal mucosa.

**Table 2:** Evaluation of the irritation potential of 100 µg/ml of SB105-A10 (21.3 µM) on the EpiVaginal tissue model.

	Viability	LDH Release	IL-1 α Release
	(%)	(Abs)	(pg/ml)
Untreated (1 hour)	100	0.9±0.1	13.4±2.9
SB105-A10 (1 hour)	81.8	0.9±0.1	11.7±4.5
Untreated (4 hours)	100	0.7±0.1	8.3±2.6
SB105-A10 (4 hours)	100.9	0.9±0.01	16.6±3.5
Untreated (18 hours)	100	1.3±0.1	33.2±4.2
SB105-A10 (18 hours)	78.5	1.1±0.01	24.5±3.0

## Discussion

We aimed to determine whether the SB105-A10 dendrimer inhibits HIV-1 infection in activated PBMCs. The present study mainly demonstrated that: i) SB105-A10 inhibited HIV replication of all of the tested reference and HIV-1 strains that were isolated from HIV-positive patients in activated PBMCs, as indicated by the HIV-1 p24 protein ELISA assay in the cell culture supernatants; ii) SB105-A10 interfered with the attachment/entry of HIV-1 by multiple mechanisms targeting cell membrane HSPGs and HIV virions; in particular, SB105-A10 bound gp41 and gp120 as revealed by the SPR analysis; and iii) SB105-A10 also inhibited the HIV infection in a biologically organotypic model of cervicovaginal epithelial tissue.

The antiviral effect of SB105-A10 was tested on a wide range of R5, X4 and dual tropic R5/X4 HIV-1 strains, and a significant and reliable decrease of HIV-1 p24 protein amount was detected by the HIV-1 p24 ELISA assay in supernatants of the activated PBMCs. Interestingly, among the HIV-1 strains that were challenged by SB105-A10, it was observed that this dendrimer was also able to suppress HIV-1 strains that were isolated from HIV-1 naïve patients and HIV-1-positive patients infected by strains resistant to certain reverse transcriptase and protease inhibitors. Hence, SB105-A10 exhibited an antiviral effect that was independent of the HIV strain tropism, with comparable IC_50_ values observed in the R5 and X4 strains.

Analysis of the antiretroviral mechanisms indicated that the dendrimer might target an early event during the viral replication. The pre-attachment and attachment assays revealed that SB105-A10 effectively inhibited the HIV infection, whereas a significant p24 protein decrease was not detected following the post-attachment procedure. These observations suggest that SB105-A10 may prevent HIV-1 infection by acting on the first phases of the viral replication cycle, which are most likely represented by viral attachment/entry. The initial interaction between HIV and the cell membrane is facilitated by interactions between the positively charged domains on gp120 and the negatively charged heparan-sulfated proteoglycans on the target cell membrane or by interactions with cell membrane lectin-binding proteins such as DC-SIGN [**47]**–[49]. Protein gp120 then binds to CD4, and this interaction induces a conformational shift of the gp120 structure that causes the formation of a bridging sheet with the exposure of the V1/V2 and V3 loop [**50]**–[52]. These steric changes allow for the binding of gp120 to the CXCR4 and CCR5 co-receptors and the fusion of the gp41 ectodomain with the cell membrane [**53]**, [54]. The fusion is determined via the formation of a six-helical bundle in which three gp41 N-terminal heptad repeats form a trimeric inner core and three C-terminal heptad repeats are packed in an antiparallel manner against the inner trimer [**55**]. The free energy that is released by the bundle formation leads to the juxtaposition and subsequent fusion of the viral and target cell membranes [**56**]. The SPR assay demonstrated that SB105-A10 bound at a high affinity to the HIV envelope protein gp41 and, to lesser extent, gp120. It is conceivable that binding of SB105-A10 to the two viral envelope glycoproteins may determine, through the dendrimer's multivalency, an alteration in the correct steric interaction between the viral envelope glycoproteins and the cellular receptors thereby inhibiting HIV-1 entry into activated PBMCs. Interestingly, the SPR indicated that the high-affinity binding between SB105-A10 and gp41 is localized to the ectodomain of gp41. SB105-A10 binds the ectodomain of gp41 in a region that encompasses the amino acids between 546 and 682. This domain is strongly involved in the formation of the bundle and plays a pivotal role in the fusion between the virus and the cell surface [**57]**, [58]. SB105-A10 might then be considered a factor that binds to both the gp41 and the gp120 envelope binding glycoproteins. It is noteworthy that several anti-HIV agents bind to the gp41 or gp120 glycoproteins. Among the agents that target gp41, T-20 is the most important and is used in antiretroviral therapy [**59**]. T-20 is derived from gp41 ectodomain sequences and prevents formation of the viral gp41 bundle, as well as the fusion and entry of HIV-1 into the target cell [**60]**–[61], by forming heterotrimers with its counterparts in gp41. The synthesis and utilization of T-20 as an antiretroviral has promoted the development of similar drugs that inhibit gp41 fusion by competitive mechanisms; in fact, C52L and HR212 suppress formation of the viral gp41 bundle and viral entry [**62]**–[63]. Conversely, other drugs have non-competitive mechanisms: RC-101, which is a cationic derivate of the defensin theta, binds directly to gp41 and suppresses the HIV infection by inhibiting HIV entry [**64]**–[65]. Several molecules bind to gp120, thereby interfering with the interaction between gp120/CD4 and/or the gp120/co-receptors. Lectins, polyanions, agents targeting the CD4-binding site of gp120 and antibodies have been described and employed in some trials [**9]**, [48], [66]–[68]. Mannose-rich glycans are present on the envelope structure and are the target of several anti-HIV molecules, such as the lectins [**9**]. Cyanovirin, Griffithsia are two of the substances, with anti-HIV characteristics, that bind to gp120 glycosylated structures and inhibit the entry of the R5 and X4 HIV-1 isolates [**69]**, [70]. Moreover, polyanions such as PRO 2000, PC-515 and cellulose sulfate recognize positively charged regions on gp120 [**71]**, [72]. PRO-2000 displays a wide range of activity by recognizing gp120, CD4 and co-receptors showing antiviral effects, especially against X4 tropic strains [**73**]. Polyanions represent a classical group of HIV-entry inhibitors; indeed two dendrimers with negatively charged branching arms (namely SPL7103 and SPL 7115) have demonstrated gp120-binding activities [**74**]. Interestingly, experiments on non human primates indicated that the intravaginally treatment of macaques by 3-5% w/w SPL7013 gel effectively prevented the vaginal transmission of SHIV89.6 chimeric virus strain [**37**]. SB105-A10 is a peptide-derived dendrimer that originates from the M6 prototype, which is a tetra-branched dendrimer [**38**]. This molecule contains a lysine core that tethers four 9-mer peptide chains [**38**] and exhibits a polycationic sequence of basic amino acids in the branching arms, in contrast with the polyanionic stretch of SPL7103 and SPL7115 [**74**]. The antiviral effectiveness of SB105-A10 is related to this sequence of basic aminoacids. In fact, the dendrimer SB104, which has a different sequence, did not show any significant anti-viral activity. Polycation substances exert opposite effects on the HIV infection; however, recently, defensin, defensin-derived (RC-101) cationic peptides and other dendrimers (polycationic viologen) have demonstrated reliable anti-HIV effects via binding to gp41 and CXCR4, respectively [**64]**, [65], [75], [76]. SB105-A10 exhibits a more complex antiretroviral activity; in fact, the SB105-A10 administration prior to an HIV challenge yields inhibitory effects on HIV replication suggesting an additional cell-membrane target. As demonstrated recently, the basic amino acids residues of SB105-A10 bind to the negatively charged sulfated/carboxyl groups of the heparan sulfate chain of HSPGs in several cell models and inhibit the attachment/entry of several viruses, such as HSV-1, HSV-2, HPV, HCMV, RSV and Ebola virus [**39]**–[41], [77]. HSPGs are cell membrane receptors composed of a core protein linked to sulfated glycosaminoglycans (GAGs). GAGs are long, negatively charged polysaccharides that are linked to HS and that are widely detectable on cell membranes of various cell types [**78**]. HSPGs are involved in the initial attachment of many viruses to the cell surface [**79**]. Examples of these viruses include Herpesviridae (HSV, HCMV), Papovaviridae (HPV), Flaviviridae (HCV, dengue), Paramyxoviridae (RSV) and Retroviridae (HIV, HTLV) [**80]**–[86]. HIV gp120 recognizes HS through V3 loop that facilitates the attachment to the host cell and subsequent infection [**86]**–[89]. Interestingly, the removal of HS or the use of heparin as a competing molecule reduced both HIV attachment and infection in several cell models such as CD4+ HeLa, T-cell lines and macrophages [**90]**–[92]. Moreover, HS are involved in the gp41-mediated fusion of HIV with the cell membrane and play an important role in the HIV infection of CD4-negative cell models (such as CD4 negative brain endothelial cells) by affecting HIV attachment and entry [**93]**, [94]. SB105-A10 consistently binds to HS in activated PBMCs. Interestingly, the activation of PBMCs by PHA induces a strong increase in heparan sulfates exposed on the cell membrane compared with unactivated PBMCs [**85**]. SB105-A10 binds to HS on the cell membrane of activated PBMCs although the treatment with either heparinase or NaCl did not completely inhibit the binding of SB105-A10 to the cell membrane, suggesting that the dendrimer may also interactwith additional cellular targets.

As reported above, SB105-A10 exhibits a broad antiviral activity against several viruses, as has been demonstrated for another dendrimer (SPL7013) that strongly inhibits the entry of HIV, HSV-1 and HSV-2 [**74**]. SB105-A10 exerts its antiviral activity against certain viruses that are transmitted by sexual intercourse; these viruses include HPV and HSV, which recognize cell targets through the viral anti-receptor binding of HS [**39]**–[41]. This characteristic indicates that SB105-A10 is a good candidate for use as a broad-spectrum microbicide that counteracts not only HIV but also HPV and HSV. It is noteworthy that the presence of inflammatory and genital lesions due to HSV and HPV infections facilitates the HIV infection transmission [**9**]. The microbicides are compounds that would protect the user from sexually transmitted infections when applied topically to mucosal structures such as the vagina and rectum. This topical prevention strategy represents a prophylactic approach that may be employed especially in individuals who do not use condoms during sexual intercourse. Interestingly, different classes of microbicides have been proposed for prevention of the HIV infection. These compounds act on various stages of the HIV mucosal infection; in fact, agents that disrupt the free virus, blocking cell capture, or target the viral structure or cell receptors or specific viral replication stages have been widely described as microbicides although the majority of these compounds have not passed the trials [**21**]. However, TDF administered orally or by gel [**23**] has yielded effective results; however, certain studies have not reproduced these results most likely because of low levels of patient compliance [**28]**, [29]. Novel evidence has also supported the use of other antiretrovirals, including combinations (TDF and emtricitabine) used in cART for oral prophylaxis, and promising results have been obtained from the initial analyses [**95]**, [96]. SB105-A10 was challenged with R5 tropic HIV-1_ada_ strain in human cervico-vaginal tissues [**64**]. The analysis of SB105-A10 antiviral activity against the R5 tropic HIV-1 strains is pivotal because the HIV-1 R5 strains are predominantly involved in the sexual transmission of HIV. The quantitative analysis of HIV-1 proviral DNA by real-time PCR demonstrated a strong decrease of the HIV-1 DNA content in the SB105-A10-treated samples compared with the untreated samples. In addition, SB105-A10 does not elicit an inflammatory response or cytotoxicity in this tissue model thus validating the potential use of this molecule as a topical microbicide. However, toxicity and efficacy studies conducted using non-human primate models must be performed to validate SB105-A10 as a topical microbicide candidate for prevention of the heterosexual transmission of HIV-1 and other specific sexually transmitted viruses.
